# The need for location-specific biometeorological indexes in Taiwan

**DOI:** 10.3389/fpubh.2022.927340

**Published:** 2022-07-22

**Authors:** Ho Ting Wong, Tuan Duong Nguyen

**Affiliations:** ^1^Department of Business Administration, College of Management, National Taiwan Normal University, Taipei, Taiwan; ^2^Department of Taiwanese Literature, College of Liberal Arts, National Cheng Kung University, Tainan, Taiwan; ^3^Department of Business Management, College of Management, National Sun Yat-sen University, Kaohsiung, Taiwan

**Keywords:** ambulance, biometeorological index, Taiwan, Taipei, weather

## Abstract

**Objective:**

As most available biometeorological indexes were developed decades ago in western countries, the benefit of using these indexes to study the effect of weather on human health in modern eastern countries is questionable. This study aimed to reconfirm the effectiveness of applying these biometeorological indexes when analyzing demand for daily emergency ambulance services (EAS) in Taipei.

**Methods:**

More than 370,000 EAS usage records were analyzed in this study. The records were first allotted into different time-series data by age, gender, triage level, and case nature (trauma/non-trauma) in order to represent different kinds of daily EAS demand. They were then regressed on biometeorological indexes [Apparent Temperature (AT) and Net Effective Temperature (NET)]; the indexes' additional descriptive power to describe the daily EAS demand over traditional weather factors was then assessed.

**Results:**

No significant difference was observed in the descriptive powers in terms of effect on daily EAS demand of the biometeorological indexes and traditional weather factors. The largest improvement on the regression models' adjusted-*R*^2^ using NET and AT was only 0.008.

**Conclusion:**

It may not be a good idea to make direct use of the biometeorological indexes developed in western countries decades ago. Taiwan should have a tailor-made biometeorological index for a better representation of its unique situation.

## Introduction

The relationship between weather and human health has long been a popular research topic among scholars in different disciplines. In the early days, scholars focused on analyzing the effects of weather on different health-related events such as mortality (1, 2), morbidity (2, 3), suicide attempts (4, 5), and emergency health services demand (6). When the quantity of related studies became saturated, scholars started to extend their research in two main directions: first, improving their data analysis techniques; and second (7, 8), replacing traditional weather factors with comprehensive biometeorological indexes as the predictors of dependent variables (9, 10). Nowadays, many different biometeorological indexes are available, including Net Effective Temperature (NET) and Apparent Temperature (AT) (11). The NET index was originally introduced based on the combined effects of air temperature and relative humidity. As its application was limited to the hotter months, the factor of wind speed was further incorporated into the index so that it could also be applied during the colder months (12). AT, meanwhile, was developed to describe the temperature and humidity perceived by humans (13). These biometeorological indexes are able to compact the information from multiple traditional weather factors into a single index. Moreover, some of them, such as Physiological Equivalent Temperature (PET), can even take into account subjective human feelings about weather conditions, as well as behavior such as clothing choice (11). Hence, biometeorological indexes are logically expected to be more capable of describing the relationship between weather and human health. They are also expected to be easier to handle than traditional weather factors during data analysis because of their single-dimensional structure.

Due to the appealing characteristics of these biometeorological indexes, they have induced a new wave of research on the relationship between weather and human health (14, 15). Although biometeorological indexes have already replaced the role of traditional weather factors as the main research focus, the comparative benefits of applying these biometeorological indexes in different countries have not been fully explored. As most of the indexes were developed decades ago, when life was quite different from that of the modern day, it is questionable whether they are up-to-date. Moreover, as the parameters for calculating these indexes from traditional weather factors were designed in western countries, the benefit of the indexes should also be reconfirmed when applying the same set of parameters in eastern countries. The above arguments suggest that biometeorological indexes could be outdated and may not be universally applicable around the world; both spatial and temporal variations could be a possible source of bias when applying biometeorological indexes in modern eastern countries.

As a result of the above issues, Wong et al. (16) examined the benefits of two biometeorological indexes by studying the relationship between weather and daily emergency ambulance service (EAS) demand. In their study, they selected NET and AT as predictors and tried to establish their relationship with daily EAS demand using multiple regression analysis. These two biometeorological indexes were selected because they are applicable throughout the whole year, unlike other indexes such as Wet-Bulb Globe Temperature (WBGT) (17) and Wind Chill Index (WCI) (18), which are only applicable to the summer and winter periods respectively. Moreover, the parameters required to calculate NET and AT only involve standard measurements that are already collected by the Central Weather Bureau of Taiwan. In contrast, when using PET, data on clothing choices and some thermophysiological factors must first be collected in order to obtain the necessary parameters to further calculate the index (11). The results of Wong et al. showed that the regression models obtained using NET and AT did not significantly outperform the models obtained using traditional weather factors. Although the authors did not reject the usefulness of the biometeorological indexes, they questioned whether the parameters of the biometeorological indexes were universally applicable. Given that their study was conducted in Hong Kong, further tests outside Hong Kong are required to confirm the results (16). Moreover, as Hong Kong and Taiwan differ in terms of weather conditions and healthcare demand, further tests could help extend the validity of the results of Wong et al. (16) beyond Hong Kong. Hence, this study aimed to reconfirm the effectiveness of applying biometeorological indexes when analyzing daily EAS demand in Taipei. EAS demand was selected because it is generally of higher volume than hospitalization and mortality. This could prevent the need to deal with low count time series data and would make the relationship between weather and EAS demand easier to examine than those of hospitalization and mortality rates (19, 20). A full understanding of the relationship between EAS demand and biometeorological indexes will help the Central Weather Bureau to forewarn the public about the impact of coming adverse weather in a more effective way.

## Methods

The data used in this study were obtained from the Taipei Fire Department; they included over 370,000 EAS usage records from April 2010 to December 2012 in Taipei. The selected dataset was the same as that used in the study by Wong and Lin (21). As that study has already been published, we adopted a dataset with the same content and time coverage so that the result of this study could be fairly compared with a recognized published study. For the same reason, all the data preprocessing steps in this study were also the same as those in Wong and Lin (21). These were: allotting the EAS usage records into different time-series data by age, gender, triage level (level 1: critical; level 2: emergency; level 3: urgent; level 4: semi-urgent; level 5: non-urgent), and case nature (trauma/non-trauma); conducting a 3-day moving average on the time series; and assuming there was a 4-day time-lag effect of weather on daily EAS demand. The 4-day lag effects for Hong Kong and Taiwan were demonstrated in related studies by Wong and Lai (22) and Wong and Lin (21) respectively. To understand the additional descriptive power of biometeorological indexes on daily EAS demand, NET and AT were selected for comparison with the use of adjusted-*R*^2^ generated by multiple regression analysis, which is the same as the method used by Wong et al. (16) in their study. Only adjusted-*R*^2^, and not the coefficient estimates of the regression models, were used to judge the usefulness of the indexes. This was because the coefficient estimates of the regression models for the indexes and typical weather factors contained different units, which implied that they were not comparable. Before conducting the regression analysis, unit root tests were conducted on all the dependent and independent variables to confirm that they were stationary time series. This was necessary because conducting regression analysis on non-stationary time series could result in inflated adjusted-*R*^2^ (23, 24). Hence, the Phillips-Perron test available in the econometric software EViews was used to conduct the unit root test, in order to ensure that all the dependent and independent variables were stationary time series. The Phillips-Perron test is different from the Dickey-Fuller test in that the test statistic of the α coefficient is modified so that serial correlation can be controlled when testing for a unit root. Basically, the Phillip-Perron test considers the equation


$$\begin{array}{l} \Delta y_{t}=\alpha y_{t-1}+\delta x_{t}+\epsilon_{t} \end{array}$$

where *x*_*t*_ are exogenous regressors, α and δ are parameters to be estimated, and ϵ_*t*_ are white noise. The existence of the unit root is based on the statistic


$$\begin{array}{l} {ℑ}_{\alpha }=t_{\alpha }{\left(\frac{\gamma_{0}}{f_{0}}\right)}^{1/2}-\frac{T\left(f_{0}-\gamma_{0}\right)\left(se\left(\hat{\alpha }\right)\right)}{2f_{0}^{1/2}} \end{array}$$

where $\hat{\alpha }$ is a parameter estimation, *t*_α_ is the t-ratio of α, s is the standard error of the test regression, γ_0_ is the error variance of ϵ_*t*_, and *f*_0_ is an estimator of the residual spectrum at frequency 0. More detailed information can be found in the EViews user manual (25).

As the equations and parameters for calculating NET and AT may not be the same across studies, the details for calculating the indexes are listed below:


$$\begin{array}{l} NET=37-\frac{37-T}{0.68-0.0014RH+\frac{1}{\left(1.76+1.4\nu^{0.75}\right)}}\\ \;-0.29T(1-0.01RH) \end{array}$$

where *T* is dry bulb temperature (°C), RH is relative humidity (%), and v is wind speed (ms^−1^).


$$\begin{array}{l} AT=-2.653+0.994\left(Ta\right)+0.0153{\left(Td\right)}^{2} \end{array}$$

where Ta is air temperature (°C) and Td is dew point temperature (°C).

Instead of the daily average values of the above two indexes, which are calculated by averaging the hourly index values of 1 day, daily maximum and minimum values were used to represent the weather on hot and cold days respectively in order to maximize the effectiveness of the indexes. The definition of hot and cold days was based on the turning point of the U-shape quadratic relationship of the daily average index value and EAS demand.

After confirming the stationarity of all the dependent and independent variables, daily EAS demand could then regress on typical weather variables and biometeorological indexes respectively and the corresponding regression models could be set out as below.

For typical weather variables,


$$\begin{array}{l} EAS\;demand=Constant+B_{1}\left(T_{avg}\right)+B_{2}T_{avg}^{2}\\ +B_{3}\left(T_{\max}-T_{\min}\right)+B_{4}\left(T_{avg}\;difference\;between\;two\;days\right)\\ +B_{5}\left(T_{avg}\;difference\;between\;three\;days\right)+B_{6}\left(RH\right)\\ +B_{7}\left(RH\;x\;T_{avg}\right)+B_{8}\left(Cloud\;amount\right)+B_{9}\left(BP\right)\\ +B_{10}\left(Precipitation\right)+B_{11}\left(Visiblity\right)\\ +B_{12}\left(WS\right)+B_{13}\left(Weekend\;and\;holiday\right) \end{array}$$

For *NET*,


$$\;\begin{array}{l} EAS\;demand=Constant+B_{1}(NET)+B_{2}NET^{2}\\ +B_{3}(NET_{\max}-NET_{\min})\\ +B_{4}(NET\;difference\;between\;two\;days)\\ +B_{5}(NET\;difference\;between\;three\;days)\\ +B_{13}(Weekend\;and\;holiday) \end{array}$$

For AT,


$$\begin{array}{l} EAS\;demand=Constant+B_{1}\left(AT\right)+B_{2}AT^{2}\\ +B_{3}\left(AT_{\max}-AT_{\min}\right)+B_{4}\left(AT\;difference\;between\;two\;days\right)\\ +B_{5}\left(AT\;difference\;between\;three\;days\right)+B_{13}(Weekend\;and\;holiday)\; \end{array}$$

All the analysis in this study was completed using SPSS 28 except for the unit root test, which was completed using EViews 12.

## Results

Table 1 shows the results of the Phillips-Perron unit root tests on all the dependent and independent variables. Significant test results (*p* < 0.001) were obtained in all unit root tests, which suggested that all the time series were stationary. Hence, they were ready for further regression analysis.

**Table 1:** Result of the Phillips-Perron unit root tests on the dependent and independent variables.

	**Data**		**Phillips-Perron test statistics**	**1% level critical value**	**5% level critical value**	**10% level critical value**
Daily EAS demand (dependent variables)	Age	0–14	−12.79	−3.44*	−2.86	−2.57
		15–34	−9.50	−3.44*	−2.86	−2.57
		35–64	−8.92	−3.44*	−2.86	−2.57
		65+	−6.46	−3.44*	−2.86	−2.57
	Gender	Male	−7.07	−3.44*	−2.86	−2.57
		Female	−9.13	−3.44*	−2.86	−2.57
	Triage level	1	−9.37	−3.44*	−2.86	−2.57
		2	−8.91	−3.44*	−2.86	−2.57
		3	−7.35	−3.44*	−2.86	−2.57
		4	−7.18	−3.44*	−2.86	−2.57
		5	−12.18	−3.44*	−2.86	−2.57
	Case nature	Trauma	−10.42	−3.44*	−2.86	−2.57
		Non-trauma	−7.26	−3.44*	−2.86	−2.57
	Overall		−8.25	−3.44*	−2.86	−2.57
Biometeorological indexes (Independent variables)	NET		−5.67	−3.44*	−2.86	−2.57
	NET_*max*_ − NET_*min*_		−26.98	−3.44*	−2.86	−2.57
	NET difference between 2 days		−76.71	−3.44*	−2.86	−2.57
	NET difference between 3 days		−37.95	−3.44*	−2.86	−2.57
	AT		−5.05	−3.44*	−2.86	−2.57
	AT_*max*_ − AT_*min*_		−20.03	−3.44*	−2.86	−2.57
	AT difference between 2 days		−40.39	−3.44*	−2.86	−2.57
	AT difference between 3 days		−27.01	−3.44*	−2.86	−2.57
Typical weather factors (Independent variables)	T_*avg*_		−6.31	−3.44*	−2.86	−2.57
	AT_*max*_ − AT_*min*_		−18.74	−3.44*	−2.86	−2.57
	T_*avg*_ difference between 2 days		−37.89	−3.44*	−2.86	−2.57
	T_*avg*_ difference between 3 days		−18.63	−3.44*	−2.86	−2.57
	RH		−12.37	−3.44*	−2.86	−2.57
	*RH* × *T*_*avg*_		−7.48	−3.44*	−2.86	−2.57
	Cloud amount		−18.88	−3.44*	−2.86	−2.57
	BP		−5.75	−3.44*	−2.86	−2.57
	Precipitation		−24.47	−3.44*	−2.86	−2.57
	Visibility		−17.77	−3.44*	−2.86	−2.57
	WS		−19.82	−3.44*	−2.86	−2.57
Controlled variable	Weekend and holiday		−25.35	−3.44*	−2.86	−2.57

* *Represent significant at this level*.

Table 2 below shows the difference between using biometeorological indexes and traditional weather factors when describing daily EAS demand. It can be seen that after upgrading the regression models from traditional weather factors to biometeorological indexes, the models' adjusted-*R*^2^ did not show any significant improvement. The largest improvement in the models' adjusted-*R*^2^ after using NET and AT was only 0.008 over the models using traditional weather factors. In particular, there were some cases where a step back was observed after using NET and AT.

**Table 2:** The difference between using biometeorological indexes and traditional weather factors in describing daily EAS demand.

	**Traditional weather factor**	**NET**		**AT**	
	***adj-R*** **^2^**	***adj-R*** **^2^**	***Change***	***adj-R*** **^2^**	**Change**
Age
0–14	n.a.	n.a.	n.a.	n.a.	n.a.
15–34	0.011	0.012	0.001	0.008	−0.003
35–64	0.011	0.011	0	0.013	0.002
65+	0.293	0.294	0.001	0.276	−0.017
Gender
Male	0.045	0.044	−0.001	0.042	−0.003
Female	0.130	0.138	0.008	0.131	0.001
Triage level
1	0.208	0.201	−0.007	0.192	−0.016
2	0.063	0.013	−0.05	0.071	0.008
3	0.051	0.055	0.004	0.052	0.001
4	n.a.	n.a.	n.a.	n.a.	n.a.
5	0.019	0.026	0.007	0.021	0.002
Case nature
Trauma	0.041	0.049	0.008	0.043	0.002
Non-trauma	0.176	0.182	0.006	0.161	−0.015
Overall	0.151	0.153	0.002	0.144	−0.007

*The factor of weekend and public holiday was controlled*.

## Discussion

The results of this study are consistent with the study by Wong et al. (16) conducted in Hong Kong, which showed that compared to traditional weather factors, relating NET and AT to daily EAS demand did not result in any significant improvement in the models' descriptive power. The benefit of using biometeorological indexes was not demonstrated in the studies, which might relate to the validity of NET and AT. As mentioned in the introduction, most biometeorological indexes were developed in western countries several decades ago; NET and AT are not an exception. Hence, the parameters for computing NET and AT may not be suitable for Taiwan in the modern era. In fact, similar research conducted in other countries also observed that there was no absolute advantage of using biometeorological indexes over traditional weather factors when studying the relationship between weather and health (26–28). Hence, clarifying the benefits of using biometeorological indexes is essential, and adopting them merely because their components look relatively complicated is definitely not a good idea.

From the temporal perspective, people are wealthier nowadays and technology is more advanced. Taking air conditioners as an example: NET and AT were first developed in the 1980s, and the ownership rate of air conditioners in Taiwan was only around 14.4% in 1980, compared to 96.0% in 2020 (29). This nearly seven-fold increase in ownership rate implies that people's resilience to extremely hot conditions is higher now than in the past because they can use their air conditioners to mitigate the heat. This further implies that the original parameters set in the 1980s may no longer be applicable in the modern world.

From a spatial perspective, Taiwan is on the line of the Tropic of Cancer, which has a significantly different climate from countries near the Equator such as Singapore. In Singapore, people do not experience a winter season. As Singapore's yearly average high temperature is 31.6°C and the record high is 36°C, it is expected that Singaporeans will have a higher ability to cope with hot days than Taiwanese people. Based on the same logic, people in countries far away from the subtropics, such as Canada, are expected to have a higher ability to cope with cold days than Taiwanese people.

The Central Weather Bureau of Taiwan has already included a biometeorological index in their weather forecast service; the index that they are using is AT (30). Following the above argument, it may not be a good idea to make direct use of the equation of AT, which was developed in the US nearly 40 years ago (13). In fact, the Hong Kong Observatory has already taken further steps to develop the Hong Kong Heat Index for enhancing the heat stress information service at the Hong Kong Observatory (31). Hence, it would be more beneficial for the Bureau to catch up with Hong Kong and to develop a tailor-made biometeorological index for modern Taiwan in order to achieve a better representation of its unique situation.

## Implications

The implications of the findings of this study are threefold, including academic, policy, and practical aspects. From the academic point of view, there is a lack of EAS-related research on Taiwan. This can be demonstrated as follows: if we use the search query of “(ambulance) AND (weather OR temperature) AND (Taiwan OR Taipei)” to conduct a search of the most popular medical journals database, PubMed, only four publications can be found that investigate the relationship between weather and EAS demand in Taiwan (21, 32–34). Hence, this study can further contribute to forming the building blocks of EAS and weather-related research for Taiwan. In the long term, a deeper understanding of the effects of biometeorological indexes on EAS demand could also help to advance the development of EAS demand forecast systems, which are already a popular subject around the world (35). An EAS demand forecast system that integrates relevant biometeorological indexes for a specific region is beneficial for preparation and planning when responding to the upcoming and emerging issues of aging population and climate change (36).

From the policy perspective, the study suggests that the current biometeorological index being used by the Central Weather Burau may not be suitable for the situation in Taiwan. Practically, it would be better for Taiwan to develop a biometeorological index that is specifically for local use. Such an index could help to formulate an extreme weather warning signal policy that would assist with making an informed decision on whether shelter services should be provided to the public. This kind of policy could help to minimize the potential impacts of extreme hot and cold weather conditions on the public. As the nature of the biometeorological index not only caters to the physical world but also to the way humans perceive weather, the direction of the abovementioned policy induced by the location-specific biometeorological index is in line with the guidelines of the “Impact-based Forecast and Warning Services” formulated by the World Meteorological Organization, which has suggested that modern weather forecasts should not only focus on the measurements of the physical world but also make impact-based predictions about what the weather will do, as this is vital to saving lives and livelihoods (37).

## Limitations and future research directions

The major limitation of this research is its geographical coverage. The discussed areas in this study only include Taipei and Hong Kong, which are both close to the Tropic of Cancer. In the future, it would be ideal to include more locations near the Equator and within the temperate zone. In this way, the conclusions could also be generalized to locations that are constantly either very hot or very cold throughout the whole year. In addition, the data used in this study is not detailed enough to make a disease-specific analysis. The reason for this limitation is that paramedics are not expected to diagnose patients. However, the limitation could be negated in the future if the data of emergency rooms could be obtained and combined with the EAS data to create a more holistic dataset with diagnoses.

## Author's note

A similar but different study was presented orally at the 2020 Management and Medical Sciences Interdisciplinary Conference. As no material was published in physical/online format, the oral presentation has not been included in the reference list.

## Data Availability Statement

The original contributions presented in the study are included in the article/supplementary material, further inquiries can be directed to the corresponding author.

## Ethics Statement

This study only analyzed anonymous EAS usage records, with no direct or indirect patient contact. The project has been certified for exemption from the Human Research Ethics Committee at National Cheng Kung University (No. 108-298).

## Author contributions

HW: participation in the design and data collection. HW and TN: analysis of data and discussion and drafting of the manuscript. All authors have read and approved the final version of the manuscript.

## Funding

This work was supported by a grant from the Ministry of Science and Technology, Taiwan (MOST 108-2410-H-110-069-MY2), and the National Taiwan Normal University (NTNU), Taiwan.

## Conflict of interest

The authors declare that the research was conducted in the absence of any commercial or financial relationships that could be construed as a potential conflict of interest.

## Publisher's Note

All claims expressed in this article are solely those of the authors and do not necessarily represent those of their affiliated organizations, or those of the publisher, the editors and the reviewers. Any product that may be evaluated in this article, or claim that may be made by its manufacturer, is not guaranteed or endorsed by the publisher.
